# Mixed Phenotype/Lineage Leukemia: Has Anything Changed for 2021 on Diagnosis, Classification, and Treatment?

**DOI:** 10.1007/s11912-022-01252-w

**Published:** 2022-04-05

**Authors:** Marie C. Béné, Anna Porwit

**Affiliations:** 1 Hematology Biology, Faculty of Medicine and Inserm, CHU de Nantes, CRCI2NA, INSERM UMR 1307 & CNRS UMR 6075 Nantes, France; 2 Faculty of Medicine, Department of Clinical Sciences, Division of Oncology and Pathology, Lund University, Sölvegatan 25b, 22185 Lund, Sweden

**Keywords:** Acute leukemia, Ambiguous lineage, MPAL, Morphology, Immunophenotyping, Genomics, Mutations, Fusion genes, Translocations, Allogeneic hematopoietic stem cell transplantation, Tyrosine kinase inhibitors, Immunotherapy, Unsupervised flow cytometry clustering

## Abstract

**Purpose of Review:**

Recent advances in the small field of the rare mixed phenotype acute leukemias (MPAL) are presented focusing on a better understanding of their pathophysiology and search for better therapeutic approaches.

**Recent Findings:**

Three aspects of respective classification, therapy, and immunophenotype of MPAL are reviewed. New proposals have been made to segregate MPAL subtypes based on their genomic landscape. In parallel, it was found that a large array of therapeutic approaches has been tested in the past few years with increasingly good results. Finally, we explored the use of unsupervised flow cytometry analysis to dissect subtle variations in markers expression to better characterize the variegating aspect of MPALs.

**Summary:**

Genomic and immunophenotypic aspects more clearly link MPAL subtypes with *bona fide* acute myeloblastic of lymphoblastic leukemias. This is likely to impact therapeutic strategies, towards a better management and outcome.

## Introduction

The unexpected co-expression of markers from different hematopoietic lineages on blast cells is often a surprise and always a challenge both in the laboratory and at the patient’s bedside. Morphology examination had long shown that some patients with acute leukemia harbored two types of blasts differing in size. The revolution of monoclonal antibodies as laboratory reagents, allowing for an extensive exploration of lineage-associated markers, also identified peculiar blasts with no definitive commitment to a given cell type, co-expressing markers of more than one lineage. The nomenclature evolved to settle, in the WHO 2008 classification of tumors of hematopoietic origin [1], on the MPAL acronym for *mixed phenotype acute leukemia*, which replaced the still much used “biphenotypic,” “biclonal,” or “bilineal” acute leukemias (BAL).

Over time, indeed, many case reports or small series of BAL were published. In the late 1990s, the European group for immunophenotyping of leukemias (EGIL) [2] proposed a scoring system that remains well-founded to date, as it made use of the now completely validated most significant lineage-specific markers. Since then, several reviews and larger series have appeared in the literature, trying to better characterize these “strange leukemias” [3, 4]. In a comprehensive review published in 2019 [5], we described a state of the art of MPAL as considered at the time. Thorough analysis of the literature confirmed the four possible immunophenotypic subtypes of MPAL (B/Myeloid (My), T/My, T/B, and T/B/My) and shed more light on their rather complex karyotypic and molecular landscape. The algorithm published by Wolach and Stone [6] also proposed a reasoned therapeutic approach that was recalled in this review paper.

Here, 3 years later, we investigated what has changed in this challenging topic. The immunophenotypic classification of MPALs, according to the lineages involved, seems now well settled. Yet, the sheer definition of MPAL seems to shrink as more and more genetic subsets of leukemia are described. Early in the definition of MPAL, chromosomal anomalies also encountered in some cases of acute myeloid (AML) or acute lymphoblastic (ALL) leukemia were recognized defining specific entities: MPAL with *BCR-ABL1* and MPAL with *KMT2A* (formerly the *MLL mixed lineage leukemia gene*) rearrangements. It has also been early recognized that some cases of “AML with recurrent genetic abnormalities” can present with an MPAL immunophenotype. This is clearly a changing concept that might have to be considered in patient management. In recent years, the therapeutic field appears the one to have progressed most substantially, still obviously on small series, but certainly with new issues to consider.

Another innovation, still not extensively explored, is the new approach of MPAL immunophenotype analysis presented here. Indeed, recently developed flow cytometry analysis tools based on machine learning or artificial intelligence could change our understanding of MPALs and modify diagnostic approaches [7•].

These three aspects are considered in this review.

## Towards a re-definition of MPAL?

The WHO classifications of tumors of hematopoietic origin have considered MPAL as acute leukemias of ambiguous lineage [1, 8]. Apart from acute undifferentiated leukemias, five entities have been delineated, considering first those with specific gene rearrangements and second those without these chromosomal abnormalities, thus classified solely based on the immunophenotype, as follows:

- Mixed phenotype acute leukemia with t(9;22)(q34;q11.2); *BCR-ABL1*
- Mixed phenotype acute leukemia with t(v;11q23); *KMT2A* rearranged
- Mixed phenotype acute leukemia, B-Myeloid, NOS
- Mixed phenotype acute leukemia, T-Myeloid, NOS
- Mixed phenotype acute leukemia, rare types (such as T/B and T/B/My).

This remains basically the current definition and classification of MPAL, with precise immunophenotypic criteria described and redefined in the most recent version of the WHO classification [8], and somewhat extended in subsequent publications (reviewed in 5).

As already mentioned, since AML with recurrent chromosomal abnormalities became singled out as separate WHO entities, it appeared that this definition would encompass some cases with MPAL immunophenotype [8]. Yet, some cases remained considered as MPAL in some series [4, 9•]. Similarly, leukemia cases with complex karyotypes or with other genetic abnormalities, which would qualify as “AML with myelodysplasia related changes” (AML-MRC), could be considered either as MPALs or AML-MRC as suggested by various authors [10•, 11•].

Besides cytogenetics, molecular analyses have become more and more frequently investigated. Several recent studies have shown that MPALs appear to display genomic anomalies also seen in AML. For instance, RUNX1 mutations seem to be observed with a relatively frequent occurrence in leukemias with MPAL immunophenotype [12] leading to a discussion of whether a case should be classified as MPAL or AML with *RUNX1* mutation (a provisional entity in WHO 2016). In a rather large series of 31 cases, Takahashi et al. [13] reported on the partition of genomic anomalies and methylation patterns, comparing T/My and B/My MPALS with AML, T-ALL, and B-ALL. They found both AML-type and ALL-type mutations in MPAL, yet with better clinical responses when the therapy was lineage-driven, in this series. More mutations were present in T/My MPALs than in B/My MPALs as well as broader methylation profiles. B/My MPALs typically presented with *RUNX1* mutations while T/My MPALs were enriched in *NOTCH1* mutations. Interestingly, analysis of the methylome showed that T/My MPALs preferentially segregate with T-ALL and that B/My MPALs preferentially segregate with AML. Similar results have been reported by Alexander et al. [9•], emphasizing the genomic heterogeneity of MPALs, not necessarily identified by morphology or even FCM. In this extensive work, mouse models permitted to better understand this variability, as well as the capacity of MPAL blasts to switch immunophenotype even in the absence of treatment, i.e., without selective pressure. By demonstrating the role of rearrangements involving ZNF384-in either B-ALL or MPAL, these authors proposed new classification entities. One would be ZNF384 leukemias, which are also characterized by an enhanced expression of FLT3, suggesting new therapeutic strategies. The other entity proposed would be that with mutations in *WT1*, i.e., WT1-mutant T/My MPALs.

Along the same lines, attempting at defining new subtypes, the work of Guttierez and Kentsis [14] suggests that acute myeloid/T-lymphoblastic leukemia (AMTL) should be segregated as a novel entity. These authors argue that this is a molecularly defined disease, based on mutations common to subsets of AML and some T-lineage ALL. Among the genes concerned are *WT1*, *PHF6*, *RUNX1* and *BCL11B.*

MPAL diagnosis thus remains based on immunophenotypic characteristics, but the complexity of the disease is increasing with the addition of molecular data to the initial cytogenetic subsets. This is likely to impact prognosis and lead to adapted therapeutic choices.

## Treatment of MPAL

Many reports indicate that the best strategy to consider when confronted with a diagnosis of MPAL is still a matter of debate [6, 15, 16••]. The multiphenotypic nature of these proliferations made it difficult to decide whether the therapeutic strategy should be that classically used for AML or for ALL. Initially, the former approach seemed to provide the best results. Yet, progress in ALL treatment, essentially with the use of pediatric schedules, ultimately yielded the best results [6, 17•]. Of interest, patients who fail to reach complete remission (CR) with such an ALL-derived regimen can still be rescued with an AML protocol. CR may however be short, and it has been advised to search for a compatible donor with a project of allogeneic hematopoietic stem cell transplantation (Allo-HSCT). Both in their 2015 and 2020 comprehensive therapeutic algorithms [6, 18••], Wolach and Stone placed Allo-HSCT as an ultimate solution, independently of the initial approach.

However, upwards from this decision, an important improvement appeared with the development of tyrosine kinase inhibitors (TKI) to block the intrinsic enzymatic activation generated by the t(9;22) translocation of the Philadelphia (Ph1) chromosome. Shimizu et al. [19] reported a similar outcome for Ph1 + MPAL patients receiving TKI and ALL patients with a Philadelphia chromosome treated with the same regimen. Imatinib and later generation TKI can thus be proposed successfully to MPAL patients with *BCR-ABL*, potentially avoiding Allo-HSCT. In a literature review, Qasrawi et al. [20•] confirmed this notion by analyzing five published studies or case reports in addition to their real-life assessment through the Surveillance, Epidemiology, and End Results (SEER) registry.

In the same review, Qasrawi et al. [20•] also confirm the poor prognosis of MPAL patients with rearrangements involving *KMT2A* (*lysine methyltransferase 2A*). Promising solutions for such patients may reside in new drugs targeting the fusion proteins involving KMT2A (reviewed in 21). Small molecules impairing the binding of menin on these proteins showed a good efficacy in blocking their leukemia-inducing properties without a negative impact on hematopoiesis [21]. Pinometostat is another small molecule that has already been tested in phase I studies. It targets disruptor of telomeric silencing 1-like (DOT1L), a methyltransferase of the histone component H3K79 involved in the leukemic properties of KMT2A fusion proteins [22]. More possibilities are explored, targeting the complex KMT2A-dependent epigenetic landscape, including the combination of several of these new drugs [23••].

Further genomic anomalies may constitute potential targets in MPAL as they do in AML. Mutations of the FMS-related tyrosine kinase 3 (*FLT3*) gene have been reported in 12–30% of all MPALs and provide a druggable target. Indeed, Andrews et al. [24] have reported two cases of T/My MPAL with *FLT3* internal tandem duplication (*FLT3-ITD*) at high variant allele frequencies (VAF). One patient responded to midostaurin, received Allo-HSCT plus sorafenib maintenance, and was still in CR 10 months post transplantation. The other one, not eligible for Allo-SCT, was still in CR with sorafenib maintenance at 14 months [24]. Tremblay et al. reported on another case of *FLT3*-mutated MPAL successfully treated with midostaurin [25].

Other recent therapeutic solutions already proposed to ALL or AML patients may also be considered suitable for MPAL patients. The membrane expression of CD19 on B/My MPAL cells theoretically represents an attractive feature to use bispecific monoclonal antibodies such as blinatumomab or even chimeric antigen-receptor (CAR) T-cells engineered to target CD19 + blasts. Some data related to this hypothesis can be found in the literature, of course scarce owing to the rarity of MPALs and to the fact that such patients cannot be enrolled in clinical trials [26, 27]. MPAL and bispecific antibodies are still an emerging option with some published information. Durer et al. [28] reported the case of a 51-year-old woman with B/My MPAL who relapsed after Allo-HSCT but reached CR2 after receiving 4 cycles of blinatumomab and 3 cycles of donor lymphocyte infusion. An extramedullary relapse was successfully treated by radiotherapy and chemotherapy and the patient was in continued CR more than 14 months after this relapse. A second case reported by Brethon et al. described a 4-month-old infant with B/My MPAL who reached CR with a combination of blinatumomab and gemtuzumab-ozogamycin and then was allotransplanted [29]. A relapse was treated with chemotherapy and midostaurin followed by CAR T-cells therapy, leading to a continuing CR of 12 months post CAR-T at the time of publication. Another case of CAR T-cell therapy was reported by Li et al. [30]. These authors used CAR T-cells prepared from lymphocytes of the donor who provided cells for Allo-HSCT when the patient relapsed after transplantation. In this intriguing story, a decrease of CAR T-cells was documented as being concomitant with another relapse that was successfully controlled by chemotherapy and a second administration of CAR T-cells from the same donor but a modified chimera. At the time of publication, the patient had been in sustained CR for 8 months with detectable levels of the second formulation of CAR T-cells.

Finally, BCL-2 targeting with venetoclax has also more recently been applied to small series of patients with encouraging results. Liu et al. [31] reported on six patients who received venetoclax with various chemotherapy regimens. All six ultimately reached CR and five proceeded to Allo-HSCT. Two patients died, one non-transplanted who progressed, and a transplanted one who developed GVHD. The other four patients were still in CR with 8-month median follow-up at the time of publication. Klocke et al. [32] reported the case of a 65-year-old man with a complicated story of recurring relapses, ultimately controlled by the administration of venetoclax and decitabine, allowing to perform a second Allo-HSCT that led to more than 1 year of sustained CR at the time of publication. Finally, two patients with bi-lineal MPAL successfully received Allo-HSCT after chemotherapy lines of treatment including venetoclax and remain in CR several months later [33].

## A New Immunophenotyping Approach

**Fig. 1:**
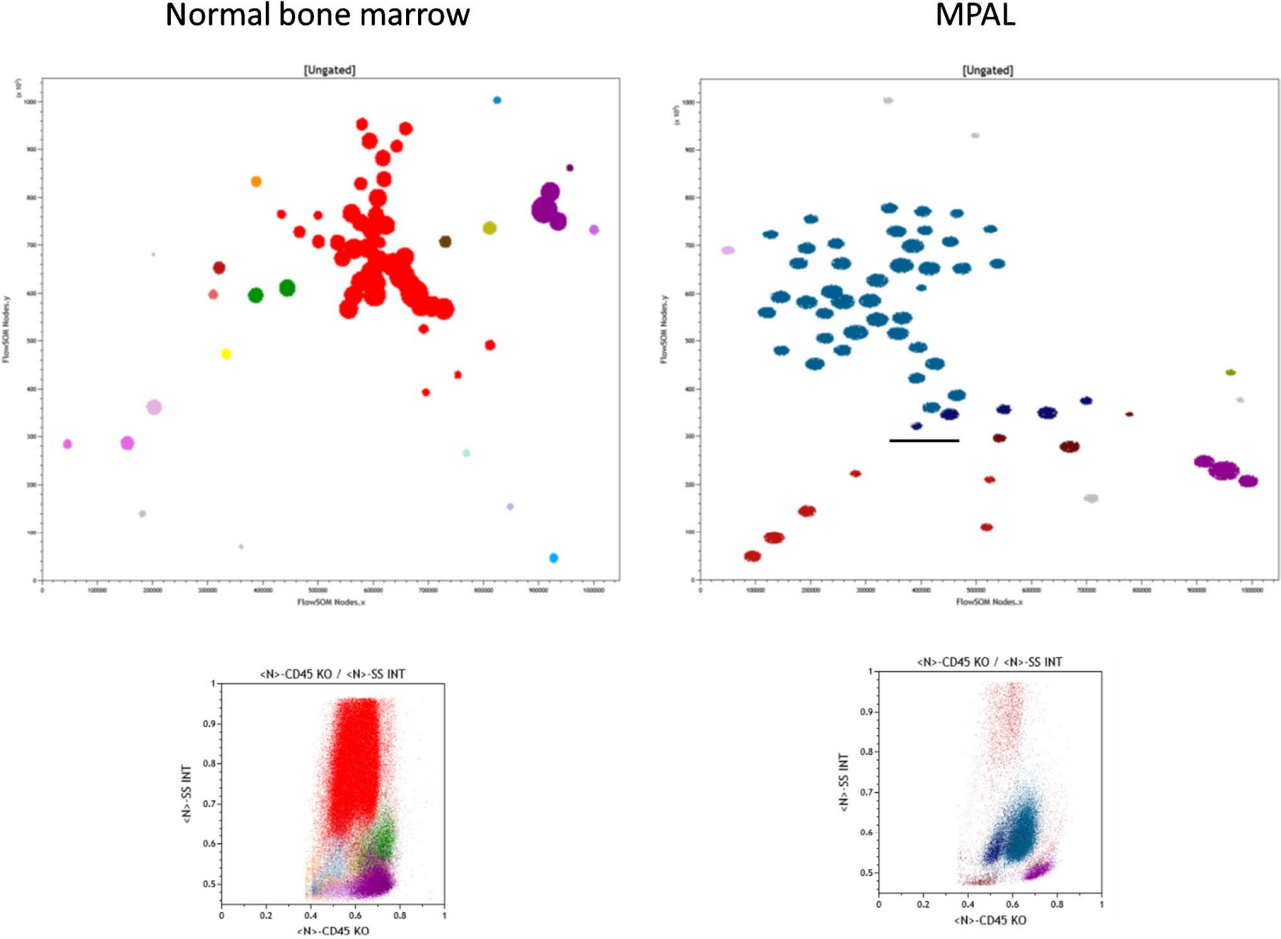
FlowSOM minimal spanning trees (MST) and CD45/SSC representations of a composite file from 10 merged normal bone marrow samples (left) and a case of MPAL B/myeloid (right). The significance of colors and characteristics of detected subpopulations are given in Table 1. A ten-color flow cytometry panel was applied on a Navios flow cytometer (Beckman Coulter) and consisted of the following antibodies: TdT-FITC, MPO-PE, CD2 ECD, HLA-DR PC5.5, CD19PC7, cyCD79a APC, CD34 A700, CD33 A750, cyCD3 BV421, CD45KO. The MSTs were created using FlowSOM after compensation check and normalization of MFI to lymphocytes as described previously (36–39)

All published reports in the literature used classical flow cytometry to analyze the immunophenotype of MPAL cases. Panels obviously differ from center to center, but basically, the markers recommended in the literature [5, 34•] have been increasingly applied, allowing for a better assessment of published series. We took the opportunity of preparing this review to explore how MPALs’ immunophenotype could be interpreted by new artificial intelligence/machine learning tools such as the FlowSOM software [35•]. The latter has been developed in the R Bioconductor environment for a fast hierarchical clustering of cell subsets with shared immunophenotypic features [7•, 35•]. Our group has published on the use of this tool for the detection of minimal residual disease in AML [36•], to characterize blastic plasmacytoid dendritic cells neoplasms (BPDCN) [37], to investigate erythroid differentiation in normal bone marrow [38] and in patients with clonal or non-clonal anemia [39]. We recently selected 10 normal bone marrow (NBM) samples analyzed with an MPAL-oriented panel (Fig. 1), the list-mode files of which were merged and then subjected to FlowSOM unsupervised clustering as previously described [38, 40]. This allowed to obtain yet another reference unsupervised definition of NBM, based on this set of lineage-associated markers. This panel uses CD45 to allow for a good delineation of the major leukocyte subsets. It also explores simultaneously the cytoplasmic markers for myeloid, B and T-lineage, myeloperoxidase (MPO), CD79a, and CD3, together with the immaturity markers intracellular Terminal deoxynucleotidyl Transferase (TdT), and surface CD34 and HLA-DR. It finally contains surface markers associated with the monocytic/myeloid (CD33), B-cell (CD19), and T-cell (CD2) lineages. In NBM, FlowSOM analysis of this panel identified 16 subsets within expected lineages (Fig. 1, Table 1). FlowSOM allowed for a more precise delineation of progenitors, granulocytes, and lymphocyte subsets than classical flow cytometry analysis. However, none of the segregated clusters expressed markers from more than one lineage. Applied to a typical B/My MPAL case of a 1-year-old girl with a hyper-triploid karyotype (78, XX, + X, -Y, + 1, + 4, + 6, + 7, + 8, -9, + 13, + 15, del (17)(p11), + 18, + 20, + 21) (Fig. 1, Table 1), this panel readily identified a large population of cells absent from NBM, i.e., MPO + CD19 + cells representing over 70% of the leukocytes, partitioned in over 40 nodes, owing to minimal variations of marker expression. In this heavily infiltrated BM, only a few granulocytes, NK, and T-cells could be observed among mature leukocytes. In the progenitor area (bermudes, 40), a small yet undescribed subset of Tdt^+^/MPO^+^/CD19^−^/CD79a^−^/DR^−^ cells represented over 6% of leukocytes. This peculiar subset could be related to the current hypothesis of leukemia-driving events at an early ill-differentiated stage of hematopoietic maturation [8].

**Table 1:** Cell subsets detected by unsupervised clustering with FlowSOM in merged normal bone marrows and a case of MPAL. Data are given as normalized mean fluorescence intensity (Cytometry A, BPDCN). *SSC*, side scatter; *Grans*, granulocytes; *NS*, non-specific. Italics in the MPAL case identify subset with true biphenotypic expression

Population	Color	TdT	MPO	CD2	HLA-DR	CD19	cyCD79a	CD34	CD33	cyCD3	CD45	SSC	%	Nodes
Merged normal bone marrow samples														
Blasts CD34 + CD19- HLA-DR +	Cyan	1.96	1.8	0.92	3.24	1.13	1.41	4.17	1.84	1.46	0.53	0.54	0.43	1
Blasts CD34 + MPO +	Blue-grey	2.18	3.78	1.2	3.66	1.24	1.29	3.08	2.47	2.11	0.52	0.6	0.23	1
Blasts CD34 + HLA-DR low	Light blue	1.37	1.46	1.05	2.15	1.08	1.13	2.59	1.22	2.36	0.51	0.48	0.3	1
HLA-DR positive only	Yellow	1.54	1.78	1.24	4.2	1.11	1.11	1.18	1.41	1.31	0.61	0.53	0.6	1
Monocytes	Green	1.64	2.83	1.09	3.15	1.03	1.08	1.06	3.62	1.49	0.72	0.6	3.07	2
Grans HLA-DR + CD45 High	Light Red	2.5	4.04	1.3	4.01	1.19	1.23	1.17	3.89	2.17	0.76	0.66	0.49	1
Grans HLA-DR + CD45 Int	Red/Brown	1.79	4.39	0.96	4.53	1.07	1.12	1.17	3.63	1.6	0.61	0.67	0.88	1
Grans	Red	1.82	3.81	0.93	1.07	1.01	1.05	1.03	1.73	1.53	0.66	0.79	78.13	41
Negative CD45 High	Brown	1.09	1.02	1.1	1.02	1	1	1.01	1.24	1.12	0.68	0.5	0.86	1
Negative CD45 low (Ery?)	Amber	1.11	1.29	0.89	1.23	1.07	1.06	1.12	1.06	1.2	0.42	0.49	0.64	1
NK-cells	Olive	1.09	1.08	3.07	0.99	1	1	1	1.01	1.15	0.7	0.51	1.13	1
T-cells (CD3 + CD2 + CD45high)	Violet	0.99	1.02	3.11	1.02	1	1	1	1	5.45	0.7	0.5	8.25	3
T-cells (CD3 + CD2-CD45low)	Light Violet	0.95	1.07	1.6	1.01	1	1	1.06	1.02	5.2	0.52	0.48	0.67	1
T-cells HLA-DR +	Dark Violet	1.03	1.16	3.42	2.61	1.06	1.03	1.02	0.99	5.31	0.74	0.49	0.26	1
B-precursors TdT +	Sea blue	4.46	1.08	1.01	4	2.72	1.57	2.37	1.06	1.05	0.41	0.49	0.3	1
B-cells CD19 + CD79a +	Helios	1.08	1.08	0.86	4.04	3.36	2.8	1.05	0.99	1.05	0.65	0.51	1.59	2
B-cells CD79a neg/dim	Pink	1.02	0.94	0.84	3.96	3.61	1.7	1.03	0.97	0.97	0.51	0.5	1.44	1
NS1-3	Grey	unspecific nodes										0.41	3	
**MPAL case**														
*Blasts 1 TdT* + *MPO* + *CD19* + *CD79a- DR- CD45 Low/INT*	*Sea Blue*	*2.73*	*3.96*	*0.98*	*1.42*	*2.16*	*1.4*	*1.03*	*0.97*	*1.14*	*0.53*	*0.55*	*3.83*	*2*
Blasts 2 TdT + MPO + CD19- CD79a- DR- CD45 Low/INT	Dark blue	2.42	2.74	1	1.48	1.8	1.28	1.02	1	1.16	0.53	0.55	6.47	5
*Blasts 3 TdT* + *MPO* + *CD19* + *CD79a* + *DR-CD45 INT*	*Sea Blue*	*2.73*	*3.85*	*1.01*	*1.88*	*3.63*	*2.96*	*1.06*	*0.99*	*1.19*	*0.64*	*0.57*	*68.31*	*40*
Grans	Red	1.46	4.5	1.02	0.78	1.07	1.07	1.02	1.14	1.24	0.61	0.94	7.03	6
Negative CD45 LOW	Brown	1.34	1.79	1.01	1.16	1.66	1.16	1.01	1	1.1	0.45	0.48	2.84	3
NK-cells	Olive	1.09	0.87	4.42	0.65	1.02	1.03	1.01	1.01	1.1	0.71	0.52	0.45	1
T-cells (CD3 + CD2 + CD45high)	Violet	1.01	1	3.8	0.66	1.01	1	1	1.01	5.26	0.7	5	8.79	3
NS1-4	Unspecific												2.07	4

In this typical case of B/My MPAL, the unsupervised clustering analysis revealed a higher number of coexisting subsets than disclosed by the classical FCM analysis. In other MPAL cases tested similarly (manuscript in preparation), this diversity appeared even more marked. This might explain the necessity to combine ALL and AML-specific chemotherapy regimens in some patients. It also provides a clue as to the evolution of some MPAL cases with so-called “lineage switch” where a very small subset of blasts present at diagnosis becomes a dominant population at relapse. Indeed, this varied landscape is likely to harbor chemoresistant subpopulations/subclones, liable to proliferate after the eradication of responding populations.

## Conclusions

In recent publications, the field of MPAL remains a niche with about 70 references in the past 3 years. Yet, the significant progress in therapeutic management is undisputable, with the progressive incorporation of modern chemo-free targeted strategies and immunotherapy. However, allo-HSCT remains a widely used solution to consolidate CR obtained with these new options, with perhaps the exception of the excellent response to TKI in *BCR-ABL1* + MPAL.

Extensive exploration of the genomic landscape of MPAL progressively highlights their complexity and often closer relationship with either ALL or AML. Whether this will lead to a change in classification/nomenclature remains a matter of debate.

This complexity is likely to be also reflected immunophenotypically with the use of unsupervised strategies of machine learning. Indeed, such approaches highlight the concomitant presence of varied types of cells unseen in normal bone marrow.

It, therefore, seems that on the three issues dealt with here, i.e., classification, treatment, and diagnosis of MPAL, the coming years will probably contribute to a better understanding of these rare diseases. The encouraging results of TKI, in *BCR-ABL1* + MPALs, for which the prognosis is now similar to that of *BCR-ABL1* + ALL let hope that other therapeutic solutions will be found. It is also predictable that they may rely on a more accurate immunophenotypic/molecular characterization and a better follow-up, in an integrated clinical/biological approach.
